# Ethanol extract of *Forsythia suspensa* root induces apoptosis of esophageal carcinoma cells via the mitochondrial apoptotic pathway

**DOI:** 10.3892/mmr.2014.2874

**Published:** 2014-11-05

**Authors:** LIANMEI ZHAO, XI YAN, JUAN SHI, FENGZHI REN, LIHUA LIU, SHIPING SUN, BAOEN SHAN

**Affiliations:** 1 Research Center, The Fourth Hospital of Hebei Medical University, Shijiazhuang, Hebei 050011, P.R. China; 2 National Laboratory of Medical Molecular Biology, Institute of Basic Medical Sciences, Chinese Academy of Medical Sciences and Peking Union Medical College, Beijing 100005, P.R. China; 3 Department of Natural Medicine Development, New Drug Research and Development Center of North China Pharmaceutical Group Corporation, Shijiazhuang, Hebei 050015, P.R. China

**Keywords:** esophageal cancer, *Forsythia suspensa* root, mitochondrial apoptotic pathway, B-cell lymphoma family, Janus kinase/signal transducer and activator of transcription 3, extracellular signal-regulated kinase pathway

## Abstract

*Forsythia suspensa* root is used in the treatment of fever and jaundice in Traditional Chinese Medicine. In the present study, the anti-tumor activity of the ethanolic extract of *Forsythia suspensa* root (FSREE) against esophageal carcinoma cells was investigated *in vitro* and *in vivo* and its anti-cancer mechanism was examined. The results revealed that FSREE, rather than *Forsythia suspensa* ethanolic extracts from the leaf (FSLEE) and fruit (FSFEE) exhibited marked anti-tumor activity towards human esophageal cancer cells. FSREE induced cancer cell apoptosis and growth arrest by downregulating B-cell lymphoma (Bcl)-2, Bcl-extra large and myeloid cell leukemia 1, while upregulating Bcl-2-associated X protein, Bcl-2 antagonist of cell death and phorbol-12-myristate-13-acetate-induced protein 1. This led to the activation of poly(ADP ribose) polymerase, caspase-3 and caspase-9, but not caspase-8. Furthermore, the anti-cancer activity of FSREE was associated with a decreased level of phosphorylated Janus kinase/signal transducer and activator of transcription 3 and extracellular-signal-regulated kinase signaling activity. It was also observed that the levels of cytochrome *c* were elevated in the cytoplasm, accounting for the loss of mitochondrial membrane potential in the TE-13 cells upon treatment with FSEER. In addition, FSEER inhibited the growth of esophageal cancer cells in xenograft models and no detectable toxicity was present in the lung or liver tissues. These observations provided further evidence of the anti-tumor effect of FSEER and may be of importance to further examine the potential role of *Forsythia suspensa* root as a therapeutic agent in esophageal carcinoma therapy.

## Introduction

Esophageal cancer is the eighth most common type of cancer, the sixth most common cause of mortality from cancer worldwide and is more common in males (1). The incidence of esophageal cancer in the high-risk northern Chinese population exceeds 100/10,0000 and it has become a significant problem in Asian populations due to its markedly poor prognosis (2,3). Although certain studies have demonstrated that the incidence of esophageal cancer is decreasing in Western countries (4), other studies have revealed that esophageal cancer has become one of the fastest-growing types of cancer in the Western world (5). Therefore, the prevalence of esophageal cancer and its poor survival rate following current therapy indicates a requirement to identify novel drugs for its treatment. The use of botanical agents or their derivatives, including isoflavone and curcumin, for the treatment of cancer has been demonstrated to be effective (6,7). *Fructus forsythia*, one of the most recognized Chinese medicinal herbs, has been widely used as an anti-inflammatory, diuretic, antidote and anti-cancer agent (8). Furthermore, a number of studies have revealed that an extract of *Fructus forsythia* fruits induces apoptosis in cancer cells, including liver, gastric and colon cancer (9), and enhances the sensitivity of cancer cells to chemotherapy (10). The root, fruit and leaf of *Fructus forsythia* have different medical uses. However, the plant part of *Fructus forsythia* with the most marked anti-tumor activity has remained to be elucidated.

In the present study, the anti-tumor activity of the root, leaf and fruit extract of *Fructus forsythia* was compared. Furthermore, the underlying mechanism of the anti-cancer effect of the ethanolic extract of *Fructus forsythia* root (FSEER) on esophageal cancer cell lines was investigated *in vitro* and *in vivo*. The ability of FSEER to inhibit the growth of esophageal cancer cells and to induce apoptosis via affecting levels of B-cell lymphoma (Bcl)-2 family proteins was examined. Furthermore, the effect of FSEER on Janus kinase (JNK)/signal transducer and activator of transcription (STAT) and extracellular-signal-regulated kinase (ERK) signaling pathways was investigated *in vitro*. In addition, the anti-tumor activity of FSEER was evaluated *in vivo* using a TE-13 esophageal cancer cell xenograft murine model.

## Materials and methods

### Reagents and antibodies

Fetal calf serum (FCS) and RPMI 1640 were purchased from Gibco-BRL (Invitrogen Life Technologies, Carlsbad, CA, USA). MTT, dimethyl sulfoxide (DMSO), RNase A and Annexin V/propidium iodide (PI) apoptosis kits were from Sigma (St. Louis, MO, USA). Monoclonal antibodies to B-cell lymphoma 2 (Bcl-2; mouse anti-rabbit; 1:1,000), Bcl-2-associated X protein (Bax, mouse anti-rabbit; 1:1,000) and GAPDH (rabbit anti-mouse; 1:10,000) and polyclonal antibodies to poly(ADP ribose) polymerase (PARP; sheep anti-rabbit, 1:1,000), caspase-3 (mouse anti-rabbit, 1:500), caspase-8 (sheep anti-rabbit, 1:500), caspase-9 (mouse anti-rabbit, 1:500) and cytochrome *c* (Cyt-*c*; sheep anti-rabbit, 1:1,000) were supplied by Santa Cruz Biotechnology, Inc. (Santa Cruz, CA, USA). Polyclonal mouse anti-rabbit antibodies targeting phosphorylated (p)-ERK (1:1,000), p-Janus kinase (JAK; 1:1,000) and p-signal transducers and activators of transcription (STAT)3 (1:1,000) were purchased from Cell Signaling Technology, Inc. (Danvers, MA, USA).

### Tumor cell lines and culture

The esophageal cancer cell lines TE-1, TE-13 and Eca-109 were obtained from the Cellular Biology Institute of the Shanghai Academy of Sciences (Shanghai, China) and the Yes-2 cell line was contributed by Professor Tagawa Masatoshi (Chiba Cancer Center Research Institute, Chiba, Japan). The cells were maintained in RPMI-1640 medium containing 10% FCS, 100 U/ml penicillin and 100 μg/ml phytomycin at 37°C in an incubator with a humidified atmosphere of the 5% CO_2_.

### Preparation of Forsythia suspensa extracts

The plant material was purchased from Le Ren Tang Pharmacy in Shijiazhuang (Hebei, China) and authenticated by Professor Fengzhi Ren (Department of Natural Medicine Development, New Drug Research and Development Center of North China Pharmaceutical Group Corporation, Shijiazhuang, China). Following drying by baking and grinding into a fine powder, the root, leaf and fruits of *Forsythia suspensa* were separated and (2 kg of each) was soaked in 95% ethanol (10 liters; Sigma) under reflux for 2×2 h. The extracts were then combined and concentrated under reduced pressure at 40°C. The ethanolic extracts of the root, leaf and fruit of *Forsythia suspensa* were termed FSEER, FSEEL and FSEEF, respectively. The concentrated extracts were then separated from the solid by filtration and concentrated using a rotary evaporator to obtain dry extracts. These were then dissolved in 100 μl ethanol and resolved with 900 μl phosphate-buffered saline (PBS) at 10 mg/ml for storage.

### Cell viability assay

The viability of treated cancer cells was determined using an MTT assay. Briefly, the cells (1×10^4^) were seeded into 96-well plates and cultured for 24 h, followed by treatment with different concentrations of the extracts for a range of durations for the different experiments. A volume of 10 μl 10 mg/ml MTT was added to each well and incubated for 3 h at 37°C, following which the supernatant was removed and 150 μl DMSO was added for 15–20 min. The absorbance was recorded using a microplate reader (Titertek Multiskan; Flow Laboratories, North Ryde, Australia) at a wavelength of 492 nm. All experiments were performed in triplicate. The effect of FSREE on tumor cell viability was detected by determining the IC_50_ value for each cell line. The effect of each extract on the proliferation of esophageal cancer cells was calculated as the percentage of cell growth inhibition using the optical density (OD) with the following formula: Inhibitory rate = ([OD control group - OD experiment group] / OD control group) ×100%.

### Flow cytometric analysis

To investigate apoptosis, 1×10^6^ cells were treated with FSREE (0.25, 0.5 and 1.0 mg/ml) for 48 h and 0.5 μg/ml FSREE for 0, 24, 48 and 72 h. The cells were collected and PBS was added to a final volume of 500 μl. The cells were then incubated with Annexin V-fluorescein isothiocyanate (FITC) and PI double stain according to the manufacturer’s instructions and analyzed using flow cytometry with a fluorescence activated cell sorting (FACS) flow cytometer (FACSIII; Becton-Dickinson, Sunnyvale, CA, USA). Data are expressed as the mean ± standard error of the mean of three independent experiments. For analysis of the mitochondrial membrane potential (MMP), 1×10^6^ cells were treated with FSREE (0.25, 0.5 and 1 mg/ml) for 48 h and then measured by labeling the cells with 1 μm rhodamine JC-1 (Molecular Probes Life Technologies, Carlsbad, CA, USA) at 37°C for 15 min and performing flow cytometric analysis.

### Isolation of cellular and cytoplasmic extracts

The cellular and cytoplasmic extract proteins were obtained as previously described (11). Briefly, cells were harvested by trypsinizing and the whole cell protein was acquired by lysing the cells on ice for 20 min in 700 μl lysis buffer with protease inhibitors and mini protease inhibitor cocktail (Roche Diagnostics, Indianapolis, IN, USA). The lysate was then centrifuged at 12,000 × g for 20 min and the supernatant was collected, and stored at −80°C. To prepare the cytoplasmic proteins, the cell pellets were suspended in 500 μl lysis buffer (see whole cell instructions) without Tween-20 detergent, samples were sonicated (1 sec × 30) on ice and then centrifuged at 10,000 × g for 20 min. The supernatant (cytoplasmic fraction) was collected in accordance with the method described in our previous report (11).

### Western blot analysis

Protein levels were evaluated using bicinchoninic acid assays (Pierce Biotechnology, Rockford, IL, USA) and 12% SDS-PAGE and were electrotransferred onto a polyvinylidene difluoride membrane (Millipore, Billerica, MA, USA). The membranes were inhibited using 5% bovine serum albumin (Sigma) for 2 h at room temperature and were incubated overnight at 4°C with the primary antibodies diluted at 1:1,000. The bound primary antibody was detected using the appropriate fluorochrome-labeled secondary anti-rabbit or mouse immunoglobulin G (IRDye 800; LI-COR Biosciences, Lincoln, NE, USA) for 1.5 h at room temperature. Following washing three times with Tris-buffered saline (Sigma) and Tween 20 (Sigma) for 10 min each, the membrane was imaged using an Odyssey infrared imaging system (LI-COR Biosciences). The levels of protein were calculated as the ratio of the intensity of protein to that of GAPDH. The experiments were performed in triplicate wells and repeated three times.

### Reverse transcription quantitative polymerase chain reaction (RT-qPCR) analysis

Total RNA was extracted from the treated cells using TRIzol reagent (Sigma) according to the manufacturer’s instructions. RT-qPCR was conducted as previously described (11), with modifications, using RT-qPCR kits from Promega Corp. (Madison, WI, USA). In brief, cDNA was prepared using RNA samples (2 μg) to which 1 μg oligo(dT), 0.5 mM deoxynucleotide triphosphate and 200 units of the Revert Aid^™^ H-Minus M-MuLV Reverse Transcriptase enzyme were added (MBI Fermentas, Hanover, MD, USA). RT-qPCR analysis was performed using primers synthesized by Sangon Biotech Co., Ltd (Shanghai, China), as shown in Table I, and 1 μl RT product was incubated with 1 unit Taq DNA polymerase in a 20-μl reaction mixture (Promega Corp.). The amplified fragments were detected on a 1.5% (w/v) agarose gel and analyzed using an IS1000 image analysis system (Alpha Innotech, San Leandro, CA, USA).

### Effect of FSEER on tumor growth in vivo

Twelve Balb/c nude mice (5–6 weeks old, 18–20 g) were purchased from the Laboratory Animal Center of Hebei Medical University (Shijiazhuang, China). The procedures using animals were approved by the Animal Care and Use Committee. The Balb/c nude mice were injected subcutaneously into the right axillary fossa with TE-13 cells (2×10^6^/0.1 ml). When the tumor growth reached a volume of ~0.1 cm^3^, the mice were randomly assigned into two groups (n=6/group). The treatment group were intraperitoneally administered FSEER (50 mg/ml) and the control group was administered an equal volume of PBS once every two days for 14 days. The tumor volumes were estimated using the following formula: 0.5 × length × width^2^, for which the length and perpendicular width were measured using calipers. Subsequently, the lung and liver tissues were stained for histological analysis using hematoxylin and eosin (H&E; Invitrogen Life Technologies) under an Olympus IX-70 microscope (Olympus Corp., Melville, NY, USA) to analyze the toxicity of FSEER.

### Statistical analysis

All data analysis was performed using SPSS 13.0 software (SPSS, Inc., Chicago, IL, USA). The statistical significance between values was determined by one-way analysis of variance, and the Student’s t-test was used to compare two independent samples. Fisher’s probability was used to analyze the difference in protein expression between groups. P<0.05 was considered to indicate a statistically significant difference. All data are expressed as the mean ± standard deviation. Results shown in the figures were obtained from at least three independent experiments with a similar pattern.

## Results

### Inhibition of cell proliferation by extracts of Forsythia suspensa

**Figure 1:**
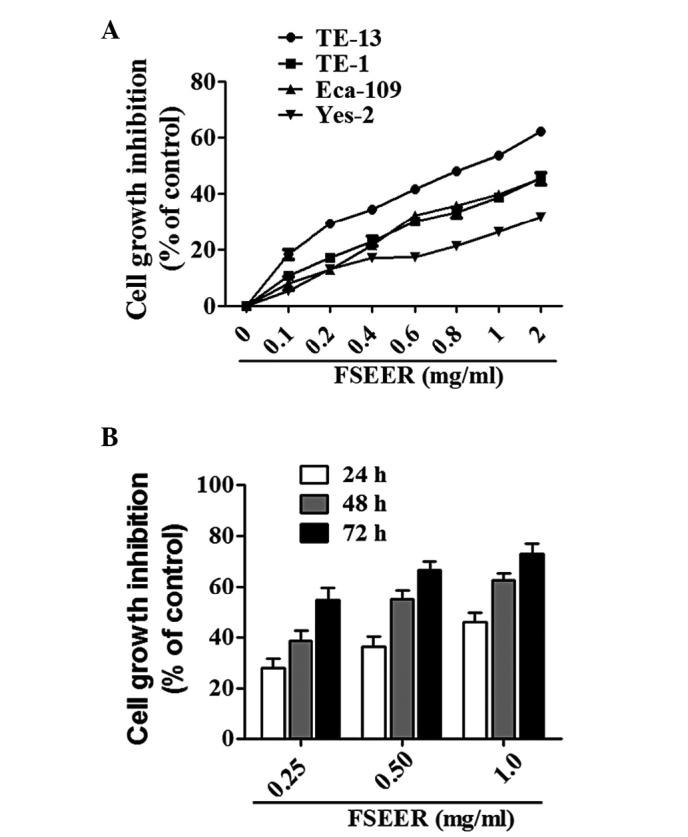
(A) FSEER inhibited proliferation of esophageal carcinoma cells in a dose-dependent manner following a 24 h treatment. (B) FSEER inhibited proliferation of esophageal carcinoma cells in a time-dependent manner. Results of the MTT assays are expressed as the mean ± standard deviation of four wells in triplicate experiments. FSEER, *Forsythia suspensa* ethanolic extract of the root.

The TE-13 cells were treated with different extracts of *Forsythia suspensa* for 48 h to evaluate their anti-cancer activity. The IC_50_ values of FSEES, FSEER and FSEEL are shown in Table II. On TE-13 cells, the IC_50_ values of FSEES, FSEER and FSEEL were 4.25, 0.58 and 78 mg/ml, respectively (Table II). This demonstrated that the extract of the root, rather than that of the fruit or leaf, of *Forsythia suspensa* had a more marked inhibitory effect on the esophageal carcinoma cells. Therefore, FSEER was selected to further investigate its inhibitory activity on esophageal cancer cells and the underlying mechanism. The inhibitory rates of 0.5 mg/ml FSEER against the esophageal cancer cell lines TE-13, ECA-109, TE-1 and Yes-2 were 64.8, 51.6, 49.0 and 48.0%, respectively (Fig. 1A). Therefore, TE-13 cells were used in the subsequent experiments. Following treatment with 0.251 mg/ml FSEER for 24, 48 and 72 h, the growth of TE-13 cells was inhibited in a dose- and time-dependent manner (Fig. 1B).

### FSEER induces cell apoptosis in vitro

**Figure 2:**
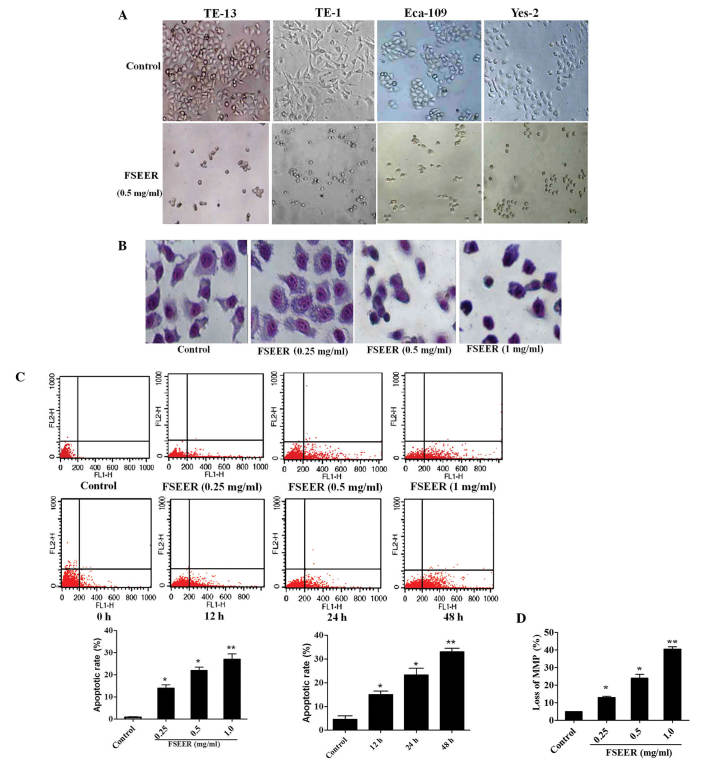
(A) Morphological changes of TE-13, TE-1, Eca-109 and Yes-2 cells treated with 0.5 mg/ml FSEER for 48 h. (B) TE-13 cells were treated with 0.251 mg/ml FSEER for 48 h and stained using Giemsa for 10 min. (C) Effects of FSEER on the apoptotic rate of TE-13 cells following a 48 h treatment. Representative histograms of Annexin fluorescein isothiocyanate-stained and propidium iodide-stained cells. ^*^P<0.05, ^**^P<0.01, compared with the control group. (D) Histogram demonstrating increased MMP by 0.5 mg/ml FSEER. ^*^P<0.05, ^**^P<0.01, compared with the control group. FSEER, *Forsythia suspensa* ethanolic extract of the root; MMP, mitochondrial membrane potential.

Giemsa staining and flow cytometry were performed to investigate whether FSEER induced TE-13 cell apoptosis. As shown in Fig. 2A and B, morphological changes observed using microscopy and Giemsa staining revealed that tumor cells exhibited decreased growth, loss of volume, cytoplasm concentration, karyokinesis and deformation to a round appearance following treatment with FSEER (0.5 mg/ml) for 48 h. However, the cells in the control group were observed to maintain a regular appearance, intensive growth and a polygonal shape. Flow cytometry was performed to estimate the rate of apoptosis by quantitative assessment of Annexin V/PI stained TE-13 cells. As shown in Fig. 2C, FSEER treatment increased the number of Annexin V-FITC-positive and PI-negative cells in a dose- and time-dependent manner compared with that in the control group. In order to determine whether FSEER-induced apoptosis of TE-13 cells was mediated through mitochondrial dysfunction, the MMP was measured using the mitochondrial-sensitive dye JC-1. As shown in Fig. 2D, the number of cells exhibiting depolarized mitochondrial membranes was significantly increased in the FSEER (0.25, 0.5, 1.0 mg/ml)-treated cells compared with that in the control group.

### Involvement of the mitochondrial signaling pathway in FSREE-induced apoptosis

**Figure 3:**
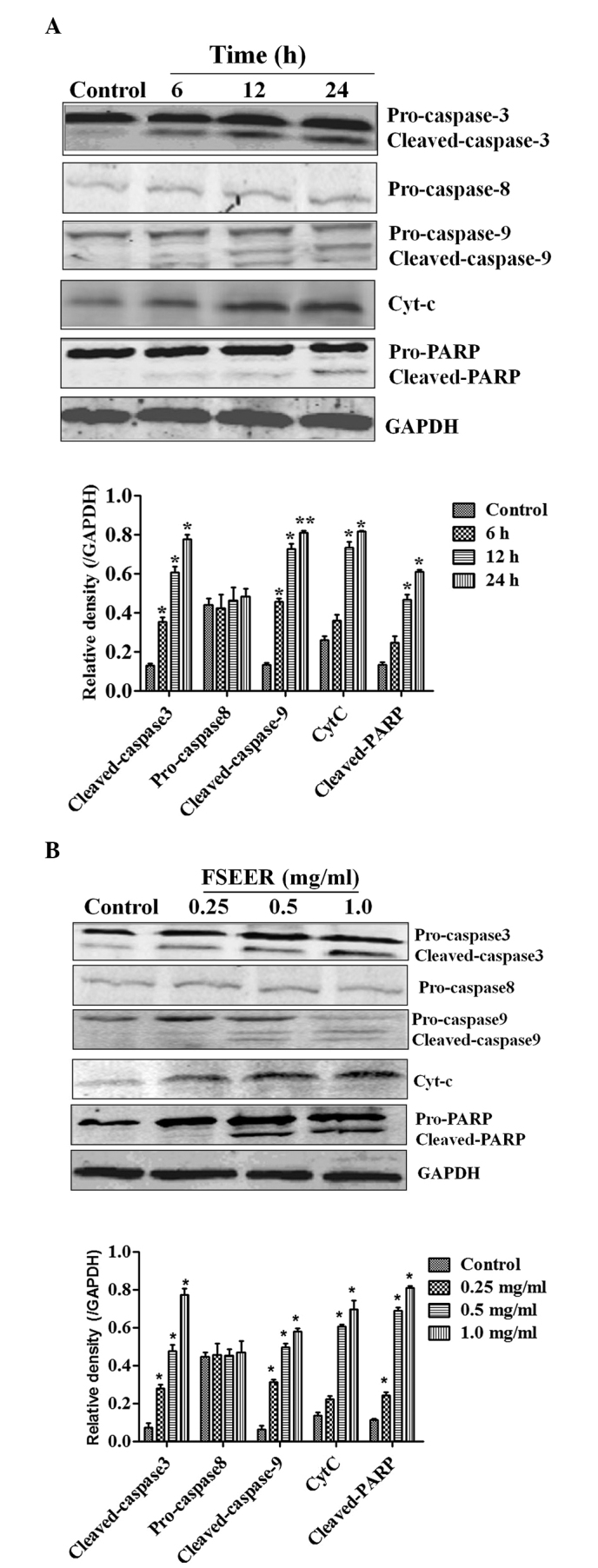
Western blot analysis of TE-13 cells treated with FSEER. The TE-13 cells were treated with (A) FSEER at various concentrations for 24 h and (B) 0.5 mg/ml FSEER for 6, 12 and 24 h. Cleaved caspase-3, caspase-9 and PARP were activated and Cyt-*c* in the cytosolic fraction increased in a dose- and time-dependent manner. ^*^P<0.05, ^**^P<0.01, compared with the control group. FSEER, *Forsythia suspensa* ethanolic extract of the root; Cyt-*c*, cytochrome *c*; PARP, poly(ADP ribose) polymerase.

Caspase-3 can be activated by a mitochondrial apoptotic pathway involving caspase-9, termed the intrinsic pathway, or by a death receptor pathway involving caspase-8, the extrinsic pathway, contributing to cell apoptosis (12,13). The results of the present study revealed that treatment of TE-13 cells with FSEER for 48 h resulted in cleavage of caspase-3, as evidenced by the appearance of 19-kDa intermediates (Fig. 3A). Furthermore, treatment of the TE-13 cells with FSEER also resulted in significantly increased cleavage of caspase-9 without changes in procaspase-8 levels (Fig. 3B). These results suggested that FRSEE triggered apoptosis through the intrinsic pathway, but not the extrinsic pathway. Activation of caspases during apoptosis results in the cleavage of critical cellular substrates, including PARP (14). Therefore, PARP has become an essential marker of caspase-3 activity in intrinsic apoptotic pathways (15). As shown in Fig. 3A, the levels of cleaved PARP fragment, which is the active form, were significantly increased following exposure to FSREE for 48 h, further confirming the activity of caspase-3 in the TE-13 cells. In addition, a key step in the intrinsic apoptotic pathway is the damage of mitochondria and the release of Cyt-*c* to activate apoptotic protease activating factor 1, which in turn activates the caspase cascade (16). Following treatment of TE-13 cells with FSEER, Cyt-*c* levels increased in the cytoplasmic fraction in a dose- and timedependent manner (Fig. 3). This result indicated that FSEER induced the release of Cyt-*c* from the mitochondria to the cytoplasm in TE-13 cells and further suggested that the mitochondrial pathway was involved in FSREE-induced apoptosis.

### Members of the Bcl-2 family are involved in FSREE-induced apoptosis of TE-13 cells

**Figure 4:**
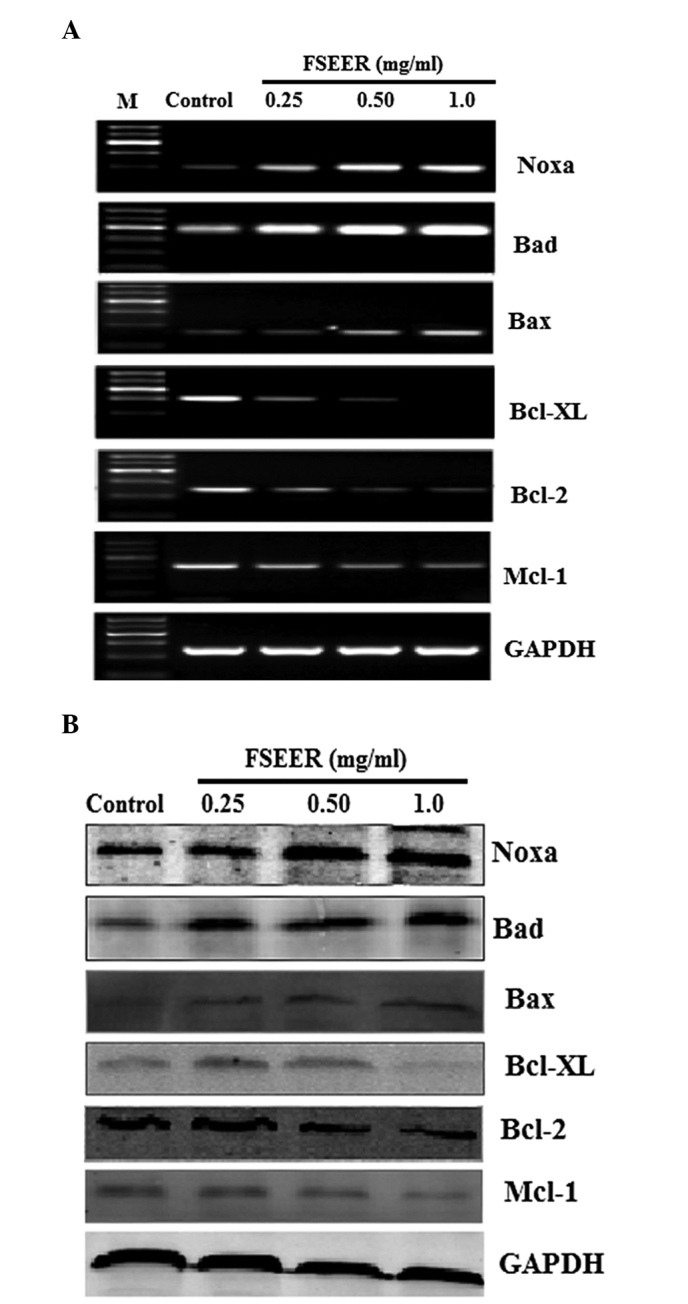
Reverse transcription quantitative polymerase chain reaction and western blot analysis of the protein expression of the Bcl2 family. TE-13 cells were treated with FSEER (0.25, 0.5 and 1.0 mg/ml) for 48 h and the (A) mRNA and (B) protein expression levels of Bcl-2, Bcl-xL, Mcl-1, Bax, Bad and Noxa were examined. FSEER, *Forsythia suspensa* ethanolic extract of the root; Bcl-2, B-cell lymphoma 2; Bcl-Xl, Bcl-extra large; Mcl-1, myeloid cell leukemia 1; Bax, Bcl-2-associated X protein; Bad; Bcl-2-associated death promoter; Noxa, phorbol-12-myristate-13-acetate-induced protein 1.

Mitochondrial integrity is regulated by the Bcl-2 family, which is constituted of pro-apoptotic members, including Bcl-2, Bcl-xL and myeloid cell leukemia 1 (Mcl-1), and anti-apoptotic members, including Bax, Bcl-2-associated death promoter (Bad) and phorbol-12-myristate-13-acetate-induced protein 1 (Noxa) (17,18). Thus, the expression of these Bcl-2 family members was detected in TE-13 cells following treatment with various concentrations of FSREE for different periods of time. As shown in Fig. 4A and B, a decrease in the expression of Bcl-2, Bcl-xL and Mcl-1 was observed, accompanied by an increase in the expression of Bax, Bad and Noxa mRNA in the TE-13 cells following treatment with FSEER (0.25–1 mg/ml) for 24 h (Fig. 4A). In addition, the change in the expression levels of the above proteins was consistent with the mRNA expression in response to treatment with FSEER (0.25–1 mg/ml) for 48 h (Fig. 4B). These results further demonstrated that the mitochondrial apoptotic pathway was activated by the Bcl-2 family in FSERR-induced apoptosis in esophageal cancer TE-13 cells.

### Effect of FSEER on the JAK/STAT3 and ERK signaling pathways

**Figure 5:**
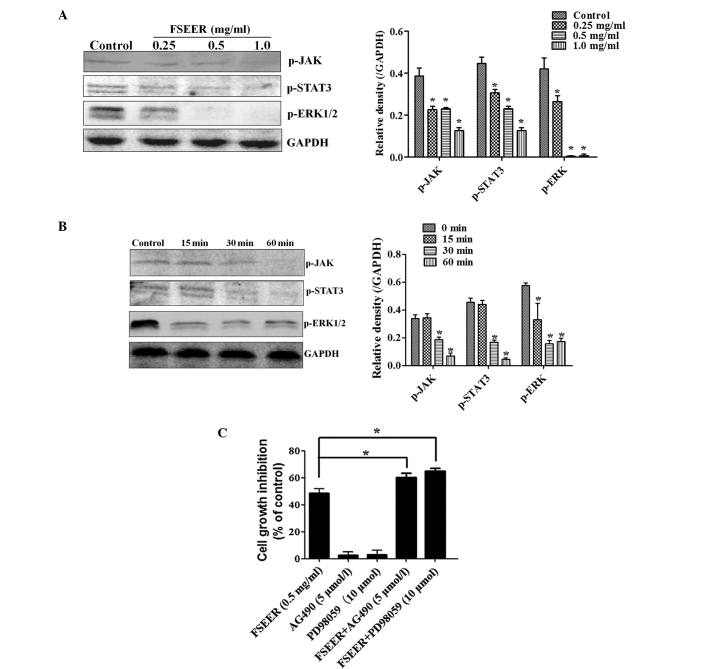
Inactivity of FSEER on the JAK/STAT3 and ERK signaling pathways in TE-13 cells. (A) TE-13 cells were treated with FSEER at 0.25, 0.5 and 1.0 mg/ml for 60 min and with (B) 0.5 mg/ml FSEER for 15, 30 and 60 min. (C) Effects of AG490 (5 mol/l) or PD98059 (10 μmol/l) alone or in combination with FSEER on the FSEER-mediated TE-13 cell proliferation. ^*^P<0.05, compared with the control group. FSEER, *Forsythia suspensa* ethanolic extract of the root; p-JAK, phosphorylated Janus kinase; STAT, signal transducer and activator of transcription; ERK, extracellular-signal-regulated kinase.

The JAK/STAT3 and ERK signaling pathways are important pathways in cell growth and apoptosis and the inactivity of these pathways may regulate the Bcl2 family resulting in growth arrest and apoptosis in certain tumor cells (19–21). Several studies have suggested that anti-apoptotic genes are regulated by interleukin 6 and STAT3, including Bcl-2, Bcl-xL and Mcl-1 (22). While these genes are induced by STAT3, the most important anti-apoptotic gene is considered to be Mcl-1 and Bcl-xL (23). The results of the present study revealed that FSEER markedly reduced the expression of p-JAK/STAT3 and p-ERK in a concentration and time-dependent manner (Fig. 5A and B), indicating that FSEER inhibited the activation of the JAK/STAT3 and ERK signaling pathways in TE-13 cells. In order to verify the involvement of these pathways in FSEER-induced apoptosis, the effect of FSEER on the proliferation of TE-13 cells was observed in the presence of an inhibitor of the signaling pathway. AG490 is a member of the typhostin family of tyrosine kinase inhibitors, which inhibit the JAK/STAT3 signaling pathway in several types of cancer cell, including esophageal carcinoma cells (24,25). Beales and Ogunwobi (26) demonstrated that the P4244 MAP kinase inhibitor PD98059 enhanced the activity of leptin-mediated esophageal adenocarcinoma cell apoptosis. The present study revealed that, although AG490 (5 μmol/l) and PD98059 (10 μmol/l) alone were not able to inhibit the proliferation of TE-13 cells, they significantly enhanced the inhibitory effect of FSEER (0.5 mg/ml) on the proliferation of TE-13 cells by ~25 and 35%, respectively (Fig. 5C). Taken together, the findings of the present study demonstrated that the induction of apoptosis of TE-13 cells by FSEER was achieved through downregulation of the JAK/STAT3 and ERK signaling pathways.

### Anti-tumor efficacy of FSEER in vivo

**Figure 6:**
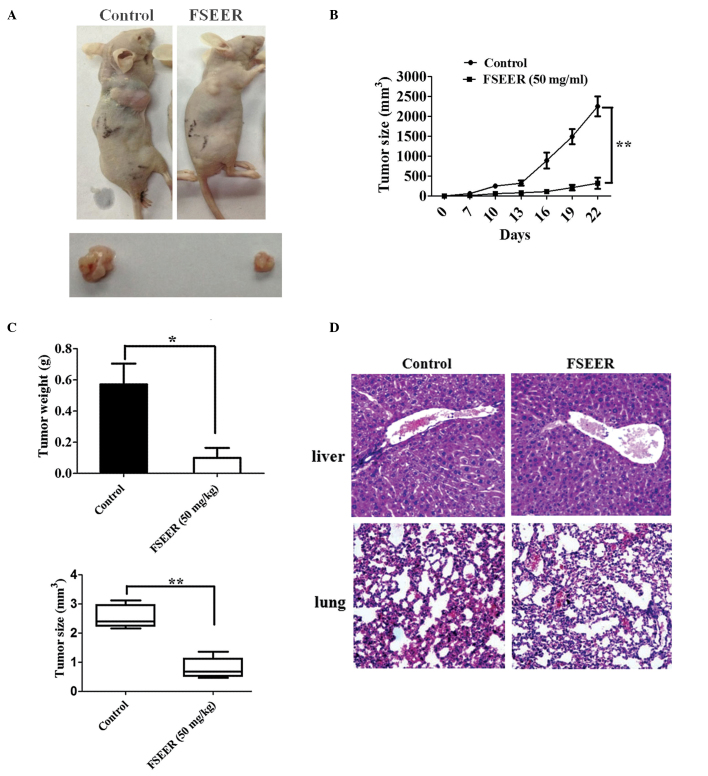
FSEER mediates the inhibition of tumor growth *in vivo*. TE13 xenograft mice were treated intraperitoneally with PBS or FSEER (50 mg/kg) once every two days for 14 days. (A) Two representative athymic nude mice from the PBS-treated and FSEER (50 mg/kg)-treated groups. (B and C) Antitumor efficacy of FSEER in the TE-13 xenograft model. Values are expressed as the mean ±standard deviation (n=5). (D) Tissues from the liver and lung of the tumor xenograft mice were stained using hematoxylin and eosin. ^*^P<0.05, ^**^P<0.01, compared with the control group. FSEER, *Forsythia suspensa* ethanolic extract of the root; PBS, phosphate-buffered saline.

The established TE-13 cells implanted into nude mice were used as a model to observe the effect of FSEER on the tumor burden *in vivo*. The treatment regimens were performed as described previously. As shown in Fig. 6A, compared with the control group, the FSEER-treated group demonstrated significant inhibition of tumor growth. Following treatment with FSEER for 20 days, the mean tumor volume of the treated group was 0.79+0.17 cm^3^ and the mean weight was 0.35+0.08 mg. These were significantly lower compared with those of the control group, which were 2.56+0.18 cm^3^ and 1.35+0.11 mg, respectively (Fig. 6B) Following inoculation of the TE-13 cells, a clear increase in tumor volume was observed from day 7 in the vehicle group until the animals were sacrificed. However, tumor volume in mice treated with FSEER (50 mg/ml) from the day of inoculation started to increase from day 10 and tumor volume increased slowly (Fig. 6C). Furthermore, no clear pathological changes were observed in the liver and lung in the H&E-stained sections of FSEER-treated mice (Fig. 6D), indicating that FSEER had no detectable toxicity in mice. In conclusion, these results indicated that FSEER exerted anti-tumor effect *in vitro* and *in vivo*.

## Discussion

*Forsythia suspensa* is used as an anti-pyretic and analgesic and is one of the essential components of Chinese Traditional Medicines used in cancer treatment. Although the leaf, root and fruit of *Forsythia suspensa* exhibit various pharmacological effects, their anti-cancer effectiveness remains to be elucidated. In the present study, the anti-proliferative effects of ethanolic extracts of leaf, root and fruit of *Forsythia suspensa* on esophageal carcinoma cells were examined. The results demonstrated that the extract of the root rather than that of the leaf or fruit produced the most marked arrest of cell growth. The present study was the first, to the best of our knowledge, to demonstrate which part of *Forsythia suspensa* is the most potent inducer of apoptosis in esophageal carcinoma cells. Of note, the leaf of *Forsythia suspensa* is commonly used for the preparation of tea in China (27) and the fruit is used for the preparation of certain oils, although these are not used for medicinal purposes (28). Previous studies have demonstrated that ethanolic extracts of *Forsythia suspensa* fruit have significant inhibitory effects against murine hepatocellular carcinoma cells (H22), human hematology cells (SMMC-7721), intestinal cancer cells (LOVo) and gastric carcinoma cells (BGC-823) (8,9). The present study revealed that FSEER inhibited the proliferation of esophageal carcinoma TE-13 cells by inducing apoptosis.

A time- and dose-dependent investigation was conducted over 72 h, with assays performed at 24, 48 and 72 h, using human TE-13 cells treated with 0.25–1.0 mg/ml FSEER. Significant growth inhibition of the TE-13 cells was observed over the entire period of the experiment compared with control cells. Morphological and flow cytometric analyses of the FSEER-treated cells demonstrated an increase in apoptotic cells, suggesting that apoptosis is important in the growth inhibitory effects of FSEER.

The activation of caspase in apoptosis occurs via two distinct pathways. Caspase 3 activation is involved in two apoptotic signaling cascades as a final apoptotic executioner. In the present study, caspase-3 and caspase-9, but not caspase-8, were activated by FSEER in the TE-13 cells, indicating that apoptosis was induced by FSEER through the intrinsic apoptotic pathway. This mechanism was similar to the role of certain chemotherapeutics on cancer cells, including paclitaxel and camptothecin (29,30), which suggested that certain compounds with anti-tumor activity were present in FSEER. The quantity of mitochondrial Cyt-*c* released into the cytoplasm is a signaling event in the intrinsic apoptotic activation pathway (31). The release of Cyt-*c* from the mitochondria into the cytoplasm supports the activation of the intrinsic apoptotic pathway. Of note, in the present study, downstream events of caspase-3, including PARP cleavage, were detected 48 h after treatment, while Cyt-*c* was observed to increase in the cytoplasm following FSEER treatment. In addition, mitochondrial outer membrane permeabilization and disruption of the MMP are independent triggers of the mitochondrial cell death cascade, resulting in the release of Cyt-*c* from the intermembrane space of the mitochondria into the cytoplasm (32). The present study demonstrated that treatment of TE-13 cells with FSREE caused rapid depolarization of MMP, depicted by representative dot blots, which demonstrated that FSEER disrupted the MMP. This accounted for the Cyt-*c* release from the mitochondria into the cytoplasm and confirmed that FSREE treatment induced TE-13 apoptosis via the mitochondrial pathway.

In addition, the Bcl-2 family, which consists of anti-apoptotic and proapoptotic proteins, are central regulators in the mitochondrial apoptotic pathway, acting to either suppress or promote the MMP changes required for release of Cyt-*c* (33,34). The Bcl-2 family has been identified as a major regulator in controlling the mitochondrial apoptotic pathway (35). The present study demonstrated that the major anti-apoptotic proteins Bcl-2, Mcl-1 and Bcl-xL were downregulated, whereas the proapoptotic proteins Bad, Bax and Noxa were upregulated in the TE-13 cells following treatment with FSEER. Therefore, the release of Cyt-*c* from the mitochondria into the cytoplasm induced by FSEER resulted from deregulation of Bcl2 family proteins.

Certain deregulated signaling pathways are involved in the occurrence and development of cancer, including esophageal carcinoma (36,37). As important signaling molecules, JAK/STAT3 and ERK are deregulated in various types of cancer cell and can phosphorylate a series of transcription factors, which regulate gene expression and are important in cell proliferation, differentiation and survival. Furthermore, the Bcl2 family is regulated mainly by the JAK/STAT3 and ERK pathways in certain tumor cells (38,39). Therefore, the present study investigated whether FSEER regulated the balance of Bcl2 family proteins via these two signaling pathways. The results revealed that levels of p-JAK/STAT3 and pERK were significantly decreased by FSEER *in vitro*, which contributed to the decreases in survival rate and induction of apoptosis in TE-13 cells. Further investigation is required to analyze whether other signaling pathways are involved in FSEER.

In the present study, the effect of FSEER on the growth of TE-13 cells *in vivo* was also assessed. The results demonstrated that FSEER (50 mg/kg) decreased the cancer burden in xenograft mice. In addition, no toxicity to lung and liver was observed at the concentration of FSEER used, which suggested that FSEER may be a potential, safe anti-cancer drug.

In conclusion, the present study demonstrated that FSEER, as an anti-tumor agent, induced the apoptosis of esophageal carcinoma cells. However, important questions regarding the inhibitory effect of FSEER remain to be elucidated, including which active components of FSEER trigger the apoptosis of cancer cells. The compounds quercetin, phillyrin and pinoresinol, which are present in the fruit of *Forsythia suspensa*, have been shown to induce apoptosis in cancer cells (8,40). However, the compounds in *Forsythia suspensea* root that exert an anti-tumor effect remain to be elucidated. Identification of these active components may assist in examining the physiological mechanisms and functions of *Forsythia suspensa* root.

## Figures and Tables

**Table I:** Primer sequences for the reverse transcription-quantitative polymerase chain reaction.

Gene	Primer sequence	Annealing temperature (°C)	Length (base pairs)
Noxa	Forward: 5′-GCTGGAAGTCGAGTGTGCTAC-3′ Reverse: 5′-CACATTCCTCTCAATTACAATGC-3′	55	211
Bad	Forward: 5′-GTTCCAGATCCCAGAGTTTGAG-3 Reverse: 5′-GGCGAGGAAGTCCCTTCTTA-3′	59	392′
Bax	Forward: 5′-CGGCGAATTGGAGATGAACTG-3′ Reverse: 5′-AGCAAAGTAGAAGAGGGCAACC-3′	62	178
Bcl-XL	Forward: 5′-ACTGGACTGTGGCATTGAG-3′ Reverse: 5′-GATTGTCGGCATACTGTTTC-3′	55	312
Bcl-2	Forward: 5′-CGACTTCGCCGAGATGTCCAGCCAG-3′ Reverse: 5′-ACTTGTGGCCCAGATAGGCACCCAG-3′	55	252
Mcl-1	Forward: 5′-TCGAGTGATGATCCATGTTTTC-3 Reverse: 5′-GATATGCCAAACCAGCTCCTAC-3′	58	302′
GAPDH	Forward: 5′-CGGATTTGGTCGTATTGGG-3′ Reverse: 5′-TGCTGGAAGATGGTGATGGGATT-3′	60	279

Noxa, phorbol-12-myristate-13-acetate-induced protein 1; Bad, Bcl-2-associated death promoter; Bax, Bcl-2-associated X protein; Bcl-2, B-cell lymphoma 2; Mcl-1, myeloid cell leukemia 1.

**Table II:** Inhibitory effect of ethanolic extract of *Forsythia suspensa* on esophageal cancer cells.

IC_50_ (mgml)	TE-13	TE-1	Eca-109	Yes-2
FSEEL	78.01	89.26	88.03	72.91
FSEES	4.25	8.92	10.90	7.82
FSEER	0.58	1.25	2.30	1.46

Each cell line was treated with the indicated extract for 48 h, respectively. All experiments were performed three times in triplicate. FSEEL, *Forsythia suspensa* ethanolic extract of the leaves; FSEES, *Forsythia suspensa* ethanolic extract of the stem; FSEER, *Forsythia suspensa* ethanolic extract of the root*.*
